# A Mixed-Methods Systematic Review of Group Reflective Practice in Medical Students

**DOI:** 10.3390/healthcare11121798

**Published:** 2023-06-19

**Authors:** Kelvin C. Y. Leung, Carmelle Peisah

**Affiliations:** 1 Research and Education Network, Westmead Hospital, Sydney, NSW 2145, Australia; 2 Discipline of Psychiatry and Mental Health, Faculty of Medicine, University of New South Wales, Sydney, NSW 2052, Australia; 3 Specialty of Psychiatry, Faculty of Medicine and Health, University of Sydney, Sydney, NSW 2006, Australia

**Keywords:** reflective practice, continuing medical education, empathy, professionalism, wellbeing

## Abstract

Background: Used primarily as a pedagogical evaluation tool for didactic teaching and skill development, reflective practice (RP) for its own merits is poorly understood. This study aimed to systematically review the literature regarding the role of group RP in fostering empathy, wellbeing, and professionalism in medical students. Methods: Electronic searches of empirical studies published between 1 January 2010 and 22 March 2022 from Medline, Embase, and PsychINFO databases were conducted. Empirical studies of any design (qualitative or quantitative) which included RP (1) involving medical students; (2) with a focus on fostering empathy, or professionalism, or personal wellbeing; and (3) provided in a group setting were included. Duplicates, non-English articles, grey literature and articles using RP to examine pedagogy and specific technical skills were excluded. Both authors screened articles independently to derive a final list of included studies, with any discrepancies resolved by discussion, until consensus reached. Articles were rated for methodological quality using the Attree and Milton checklist for qualitative studies; the Oxford Centre for Evidence-Based Medicine criteria, and the Alberta Heritage Foundation for Medical Research Standard Quality Assessment Criteria for quantitative studies. Results: Of 314 articles identified, 18 were included: 9 qualitative; 4 quantitative and 5 mixed methodology. Settings included United States (6), United Kingdom (3), Australia (3), France (2), Taiwan (2), Germany (1), and Ireland (1). Themes were (i) professionalism: bridging theoretical paradigms and practice; (ii) halting empathy decline; (iii) wellbeing: shared experience. Additional themes regarding the “successful“ delivery of RP groups in facilitating these outcomes also emerged. Conclusions: This first systematic review of group RP in medical students shows that RP may bring theory to life in clinical dilemmas, while fostering collegiality and mitigating against isolation amongst students, despite the absence of studies directly examining wellbeing. These findings support the value of RP integration focusing on emotive and humanitarian processes into contemporary medical education for medical students. Systematic review registration: PROSPERO CRD42022322496.

## 1. Introduction

Reflective practice (RP) has long-been a tool in medical education [1]. With broad origins across sociology, management and organisational learning dating back to the 1930s, the term RP lacks clarity in definition [2]. This lack of clarity is the culmination of almost a century of an evolving RP paradigm based on a range of learning experiences involving both cognitive processes (i.e., thinking about the experience) and emotive processes (i.e., feeling about the experience). This fittingly explains its inherent and diverse use in medicine. For example, as applied to medicine, RP may be defined as a process of self-questioning and experiential learning involving recapturing and evaluating clinical experiences to promote self-understanding, professionalism, “practical wisdom” and ideally, patient-care [3]. At its essence, the use of RP is the application of experiential learning to inform and influence future outcomes and practices [2]. As such, in the context of medical practices, these outcomes relate to, and ideally benefit, both patients and doctors alike.

Best undertaken as guided or facilitated [1], group RP is an efficient mode for facilitated delivery. Notably, key to many of the original conceptualisations of RP was that it was based on individual learning through a collective means, namely by observation of, and learning from, others [2]. The use of group facilitation of RP is exemplified by the Balint group, a particular type of reflective practice where clinicians meet regularly to discuss cases from their practices, with a focus on emotional content and clinical interactions [4].

Intuitively, therefore, practising reflection early and nurturing it longitudinally throughout one’s medical career would appear to be particularly useful in regards to fostering professionalism and the humanitarian aspects of medicine (e.g., respect for others, integrity, duty, honour, altruism, accountability and excellence) [5]. The importance of bringing these “soft skills” out of the hidden curriculum of medical training and into the open curriculum has not been lost on medical educators [5]. Notwithstanding these instinctive benefits of RP, actual empirical investigation of the benefits of RP for fostering humanitarianism is extremely limited. Notably, a redesigned integrative curriculum incorporating group RP at Harvard Medical School has shown a long-lasting improvement in psychosocial, relational and humanistic attitudes in medical graduates, even when confidence in managing patients with psychosocial problems and practising humanistic medicine was evaluated 10 years later [6].

Beyond this curriculum examination, RP has been evaluated only as a pedagogical evaluation tool, for example, to ask learners the question: how did you learn communication skills [7], first-year pathology [8], case-solving skills [9] or procedures [10]? While the use of RP for such technical skill acquisition is an important learning process for medical students, we know little about its use in fostering more emotive, abstract processes intrinsic to medical professionalism. This unexamined use is also more aligned with its original intended purpose [2]. In particular, the role of RP in promoting professionalism, empathy, as well as emotional self-benefit, have not been reviewed. The latter benefit is of particular relevance in the wake of the COVID-19 pandemic and its effects on wellbeing of medical students [11,12], who are often neglected in the wellbeing space. Further to our earlier comments about lifelong learning starting in medical school, and as deliverers ourselves of an RP programme for medical students, we sought to examine the evidence in this population.

This highlights an untapped evidence-base regarding the value of RP beyond its use to augment technical skill-based learning. This systematic review aims to explore the literature regarding the use of group-based RP to enhance empathy, professionalism and personal wellbeing amongst medical students. The modified research PICo (Population, Interest, Context) question [13] is: *What is the role of group reflective practice in promoting empathy, professionalism and wellbeing in medical students?*

## 2. Materials and Methods

### 2.1. Design, Protocol, and Registration

Preferred Reporting Items for Systematic Reviews and Meta-Analysis (PRISMA) recommendations were used as a framework for this review [14]. The protocol for this systematic review was registered on PROSPERO (ID: CRD42022322496).

### 2.2. Search Strategy

Electronic searches of databases, including Medline, Embase, and PsychINFO, between 1 January 2010 and 22 March 2022 were conducted using search terms designed to identify studies reporting the use of any forms of group RP in medical students. The restriction to 2010 was chosen because we aimed to capture approaches which reflect contemporary educational practice and context.

Search terms were determined using an iterative process by identifying common terminology used in the literature to cover the three key domains (empathy, professionalism and wellbeing) in our research question. For professionalism, despite its lack of clarity in definition, we used concepts of ‘clinical competence’, ‘patient-centred care’ and ‘doctor-patient relationship’ in our search terms as they were the most recursive in our preliminary searches. In terms of operationalising wellbeing, we utilised the most studied construct: ‘burnout’, as a search proxy for wellbeing.

The following combination of terms were used:

1. ‘reflection’, or ‘reflective practice’, or ‘reflective thinking’, or ‘reflective learning’, or reflective group’, or ‘balint group’, AND
2. ‘group’, AND
3. ‘medical students’, AND
4. ‘empathy’, or ‘clinical competence’, or ‘patient centred care’, or ‘communication’, or ‘doctor patient relationship’, or ‘burnout’.

### 2.3. Study Selection

#### 2.3.1. Inclusion Criteria

The published peer-reviewed literature was reviewed. Empirical (original) studies, of any design (qualitative or quantitative) which included RP (1) involving medical students; (2) with a focus on fostering empathy, or professionalism, or personal wellbeing; and (3) provided in a group setting, were identified.

#### 2.3.2. Exclusion Criteria

Excluded articles included commentaries, literature reviews, meta-analyses, editorials, letters or grey literatures. Articles were also excluded if (1) the content did not have a component of reflection; (2) RP was used as a pedagogical evaluation tool for medical education, curriculum or didactic programme (e.g., improving delivery and/or design of these programmes); focused on specific technical skill acquisition (e.g., procedural skills, communication skills, clinical reasoning, and diagnostic competence); (3) RP was undertaken as a solo exercise; or (4) they were non-English articles.

### 2.4. Review Team

The review team comprised experienced systematic reviewers, with qualitative and quantitative research experience, as well as being content experts responsible for delivering RP groups. The lead author, an advanced psychiatry trainee with expertise in delivering RP groups and undertaking systematic reviews, undertook the database searches, screening and integration. The second author, who is a senior psychiatrist with extensive experience undertaking qualitative research and systematic reviews as well as delivering RP groups, assisted with screening and the thematic analysis.

### 2.5. Screening and Data Extraction

Database searches were performed, validated and short-listed by the first author. The short-listed abstracts were screened by both authors to determine their eligibility against the inclusion and exclusion criteria. Any disagreements were discussed to reach consensus. For abstracts meeting inclusion criteria, full-text articles were then obtained for further screening performed by both authors working independently to derive a final list of included studies, with any discrepancies resolved by discussion until consensus reached.

### 2.6. Quality Assessment

Qualitative studies were rated using Attree and Milton’s checklist (2006) [15]. This checklist included criteria for rating methodological quality such as research context and background, aims and objectives, study design, sampling, data collection, results analysis, reflexivity, study value and ethical considerations. Each checklist domain was rated from A (no or few flaws) to D (significant flaws threatening the study validity), with the final quality score (A–D) determined by majority grade across domains.

Quantitative studies were appraised using Kmet et al.’s Alberta Heritage Foundation for Medical Research Standard Quality Assessment Criteria (2004) [16]. The checklist provided operationalised criteria including objectives, appropriateness of design, selection of subjects, random allocation and blinding, exposure and outcome measures, sample size, analytic methods, estimates of variance, confounding, results reporting and conclusions, with a final rating score expressed as percentage of the maximum total score. While there is no established score-based rating for overall quality, other systematic reviews have defined >80% as high quality [17,18].

Levels of evidence for quantitative studies were rated using the Oxford Centre for Evidence-Based Medicine criteria (2011) [19]. For interventional studies, level 1 includes systematic reviews of randomised or n-of-1 trials; level 2 includes randomised trials and observational studies with dramatic effect; level 3 includes cohort studies; level 4 includes case-controlled studies, case series, or historically controlled studies; and level 5 is mechanistic reasoning. Level may be graded down based on methodological flaw or small effect size. Qualitative studies are not considered for these criteria.

Both authors independently scored all included papers for quality according to the above criteria with scoring differences discussed until consensus reached.

### 2.7. Data Analysis

A table was created to extract relevant data, including author details, year of studies, country of studies, study aims, characteristics of participants and settings, study design, comparison group(s) (if any), outcome measures, limitations, level of evidence and aspects of methodological quality and score (see Appendix A). Both authors reviewed the data synthesis.

The heterogeneity of studies and inconsistent use of measures meant that the data collected in this review were unsuitable for quantitative synthesis or a meta-analysis [20].

We used a convergent integrated approach for this mixed-methods systematic review, combining both forms of data (i.e., qualitative and quantitative) into a single mixed methods synthesis, codifying both forms of data using thematic analysis. As such, data was synthesised qualitatively using inductive thematic analysis to identify salient themes. Differences and similarities across the data set were revealed using an iterative, constant comparison method [21]. First, the data was coded separately by both authors, looking for emerging themes from the included papers. Second, both authors discussed the codes and jointly re-coded potentially unclear ones. Reflexivity was considered at every step from data collection to thematic analysis. It is worthy to note that, as previously stated, both authors were involved in the delivery of medical student RP groups, and, as champions of the technique, were perhaps positively favoured towards its delivery and its justification. Finally, all findings were critically tested and discussed to resolve any discrepancies.

## 3. Results

### 3.1. Search Results

**Figure 1:**
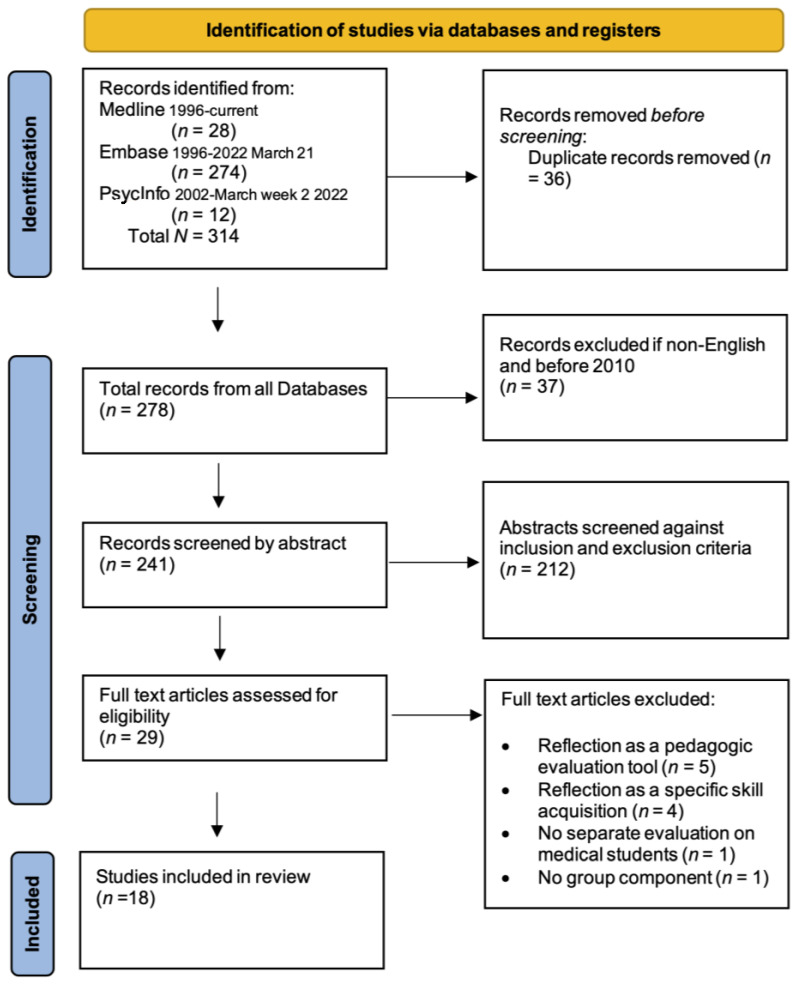
PRISMA flowchart of the results of the systematic review.

From an initial 314 records identified, 18 studies were included in the review, as per the PRISMA flow diagram (Figure 1).

### 3.2. Study Characteristics

Of 18 studies included, six were from United States [22,23,24,25,26,27], three from United Kingdom [28,29,30], three from Australia [31,32,33], two from France [34,35], two from Taiwan [36,37], one from Germany [38] and one from Ireland [39].

Nine used qualitative methodology [23,27,29,31,32,33,36,37,38], four used quantitative methodology [26,30,34,35] and five used mixed methodology [22,24,25,28,39].

Study settings were all based in universities or medical schools from metropolitan or urban areas in which students were doing their clinical years, except for Gold et al. (2019) [25], which recruited first- and second-year medical students, whose clinical experiences were unknown. 12 studies included RP groups integrated as part of a clerkship, curriculum, or certificate [22,23,26,27,32,33,36,39], while 8 others involved formal delivery of RP groups as a trial either on its own or as part of a program [24,25,28,29,30,31,34,35,38].

The characteristics, summary findings, quality ratings, and level of evidence for each included study are summarised in Table A1 (see Appendix A).

### 3.3. Quality and Bias Analysis

Of 14 studies utilising qualitative methodology, three were rated ‘A’ [27,28,38]; six were rated ‘B’ [24,29,33,36,37,39]; and five were rated ‘C’ [22,23,25,31,32] using Attree and Milton’s ratings (2006).

Of the nine studies using quantitative methodology [22,24,25,26,28,30,34,35,39], quality ratings ranged from 27% to 93% using Kmet et al.’s criteria (2004). Level of evidence based on OCEBM (2011) ratings included two at level 2 [34,35]; five at level 3 [22,24,26,28,30]; and one each at levels 4 [39] and 5 [25].

### 3.4. Synthesis of Results

The thematic analysis generated a number of themes elucidating links between group RP and professionalism, empathy and wellbeing in medical students:

#### 3.4.1. Professionalism: Bridging Clinical and Theoretical Paradigms to Serve the Doctor Patient-Relationship

Six of the studies demonstrated that RP cultivates professionalism in medical students by bridging theory and practice in relation to the doctor-patient relationship [29] and the biopsychosocial context [36]. Often in relation to dilemmas in clinical practice and complex patients [27], reflections can be triggered specifically in relation to older patients [28], patients with borderline personality disorder [34], and clerkship challenges [23]. These studies illustrated the value of RP in facilitating the emotional component of patient interactions and the humanitarian aspects of professionalism. What was notable was the role of formal structured RP, namely Balint groups, in educating students about these emotional aspects of the doctor-patient relationship [29,34].

In a RP group, students can bring in complex cases and clinical dilemmas [27], often not being immediately clear to them the nuance of the patient contact, clinical context, and/or psychosocial factors, which may interplay with their emotional and personal experience in relation to these cases and beyond [23,25,29,36]. Moreover, Bird et al. emphasised the importance of creating a setting that is conducive to “comfortable reflection” [22]. These cumulative elements appear to be key to assist medical students make sense of the theoretical bases behind their clinical encounters and contextualise these humanistic interactions. Five studies specifically addressed that having awareness to the doctor-patient relationship is akin to medical professionalism [28,29,31,32,33].

#### 3.4.2. Empathy: Halting Empathy Decline

Six studies examined the effect of RP on empathy preservation as demonstrated by Jefferson Scale of Empathy [24,26,35,39], modified Emotional Self-Awareness Scale [25] and the empathic approach in response to case reports [34].

Three studies demonstrated a significant improvement of self-administered empathy scores after RP groups [26,35,39]. A notable finding was that empathy or the ability to tolerate diverse perspectives was perceived to decrease over time [34], but RP groups may have a place in preserving students’ empathy [24], or even improving empathic ability throughout the course of patient care [25]. RP groups may also provide opportunities to contextualize empathic responses. Again, Balint groups may be the key to this [34,35]. One study, in particular, utilised 10 two-hour weekly Balint sessions to facilitate enhanced empathic approaches within the context of the actual doctor-patient relationship, rather than promoting more general empathic responses [34].

#### 3.4.3. Wellbeing: The Value of Shared Experience

No study directly measured wellbeing. However, one RP embedded within a curriculum used RP to promote resilience, helping students deal with setbacks and challenges experienced during clinical training, while also finding meaning [22]. Four studies demonstrated that group practice specifically facilitated connectedness and offset isolation in medical school [22,24,25,37], providing a safe environment for mutual support and shared experiences, as well as allowing exposure to and tolerance of diverse perspectives [25,37].

On the other hand, two studies found that some medical students felt unable to share their voice at times, restrained by feared repercussions of opening up in hierarchical environment [27,38]. This was even in the face of potentially inappropriate or harmful practices observed.

These indirect but potentially positive effects on student wellbeing mediated by RP groups appeared to be contingent upon providing a “comfortable” environment for reflection [22].

As such, other important additional themes emerged from the review in relation to the practical and successful delivery of RP groups:

#### 3.4.4. Ingredients for Successful RP Groups

A number of factors were identified that may either enhance or detract from the “success” of RP groups. For example, the various benefits of voluntary [22,29,31] versus compulsory attendance [32,38] have been explored. Voluntary, unforced participation appears to foster a sense of safety and collegiality in the group. These participants may predispose to benefits from the process. However, without the need to attend compulsorily students may rather resort to their familiar learning methods, missing the benefits of group RP. The challenges of making reflection relevant to medical students were reported, with some students failing to see any relevance from such reflection for either their work as students or physicians [22,32,38], suggesting that the utility of such groups may be contingent upon the psychological mindedness of the particular cohort. Brand et al. (2016) explored the value of “pre-reflection” to facilitate delivery and engagement of students [28]. Timing of groups [31], when students have sufficient clinical experience, or specifically, under general practice settings [32,33] to render such groups more meaningful also appears to be essential. Highlighting the importance of safe space [22,24], some students encountered interpersonal problems that impeded openness to engage. Special adaptation of group RP or Balint group to medical students and maintaining a dynamic approach responsive to the needs of a particular cohort are hence key elements. However, a pragmatic component of success is ultimately finding enough enthusiastic and skilled facilitators to run such groups [38].

#### 3.4.5. Innovative Delivery Methods

A range of innovative RP delivery methods have been examined including (i) the Depth of Field reflective learning resources which uses photo-elicitation techniques, older adults’ narratives and collaborative dialogues in the classroom [28]; (ii) resilience skills curriculum which employs core virtues of positive psychology, including intellectual strengths, interpersonal strengths, and temperance strengths [22]; (iii) the VALOR program which involves peer groups of balanced demographics such as gender, preferences for clerkship order, and prior experiences with other students in the cohort [23]; and (iv) RP embedded within the Student Psychotherapy Scheme (SPS) which provides early opportunities for using psychodynamic psychotherapy and student practice of such to teach doctor-patient relationships and reflection [30].

Further, responsive to recent COVID-imposed exigencies, online forums for RP delivery have been found acceptable by students [24] as have combined group and written reflections [28].

## 4. Discussion

As far as we are aware, this is the first systematic review to capture the evidence base regarding the use of group-based RP to enhance empathy, professionalism, and personal wellbeing amongst medical students. We note that, additionally, other important themes emerged in relation to putative ingredients for a “successful” group as well as innovative delivery models for RP.

Albeit tentative on the basis of a mixed-methods review of studies of variable quality, our findings suggest a range of potentially important learning outcomes of RP in relation to more humanitarian aspects of medicine. For example, when delivered “successfully” (and we as yet do not know what this means), RP may further student understanding of the biopsychosocial context of patients, in particular, conceptualising patients as individuals. Although first proposed by Engel (1977) [40], the biopsychosocial model still remains relevant to medical teaching today as a means of promoting student understanding of the patient’s subjective experience and context, and the effect of psychosocial variables on illness [41].

Promoting understanding of the biopsychosocial context of patients in turn enhances empathy. It is therefore not surprising that there was striking evidence in relation to the effects of RP on empathy borne out in the quantitative studies reviewed [24,25,26,34,35,39]. This is particularly important for more complex patients (often referred to as “heartsink patients” [42]) and also for older patients whose context is often not well-understood by students [28,43]. This has implications for understanding the doctor-patient relationship, and in particular, the resonance or distress associated with specific clinical contexts [27,34]. Such understanding can be akin to psychotherapy concepts of countertransference rendering it not surprising that RP delivery within a psychotherapy teaching programme may bring important insights [30,44]. Understanding the emotive responses to patients is dependent on a process of self-reflection, and awareness of the potential in patients to generate unexpected reactions within the treating clinician. These insights are aligned with the aforementioned benefits of RP [2] and go beyond the more technical skill learning of history taking, use of open questions and other communication skills.

Many of the learning outcomes described here underpin professionalism, which, although is a very broad multidimensional construct, comprises both humanitarianism and the capacity to think critically and reflectively about the doctor-patient relationship in primary service of patient welfare [5,45]. If RP is indeed “instrumental in developing professionalism” then there is an imperative to optimise its teaching [46]. This is all the more so given that the teaching of professionalism is often neglected in medical curricula or relegated to the aforementioned hidden curriculum [47].

We noted that despite the deliberative search based on a commonly used proxy for wellbeing, we found no studies directly examining links either between RP and burnout or in fact any other direct measure of wellbeing. Notwithstanding this, we did observe that RP facilitated connectedness, support and sharing of experiences while mitigating against isolation. Much of these findings appeared to be contingent on the way the RP groups were delivered, bringing us to the salience of some of the additional themes that emerged.

Notwithstanding the lack of operationalisation of what constitutes a “successful” RP group, as educators delivering such groups ourselves, we found some of the additional themes that emerged from the data illuminative from a practical perspective. First, the timing of delivering RP within an undergraduate medical curricula programme is important, and echoed by others seeking to teach professionalism who recognise the need for it to be contextualised during clinical placements to ensure experiential learning and to avoid these important concepts being lost amongst the basic sciences and rote-learning of factual knowledge [48].

Secondly, while there seems to be little doubt as to the importance of the collective experience of RP groups from a learning perspective as stated earlier [2], we would add from the data here that the collective learning of shared emotional experience must be undertaken in a “safe space”. Hearteningly, given the explosion of virtual learning since the COVID pandemic, such a safe space might indeed be achieved in a virtual environment [24].

Thirdly, another question in relation to success arises as to the issue of voluntary versus compulsory groups, particularly relevant to medical educators setting up curricula. Lack et al. (2019), in evaluating whether a compulsory reflective group activity enabled constructive sharing of emotions, noted that students–amongst whom 82% wished to repeat the group RP experience again in their training–reported positive learning experiences, echoed by the facilitators [49]. The non-judgmental format and facilitation with guidance, relevancy and feedback shed light on the potential of structured, collective, and perhaps guided reflection in the educational context. Notwithstanding the finding of this single study, we consider that the issue of compulsory versus non-compulsory groups remains unresolved. Do we focus on the converted, facilitating reflection amongst engaged medical students, who seek to gain the most but perhaps are the least in need of RP? Or do we try to achieve some reflection amongst all students, some of whom will be disengaged or withdraw from groups?

Finally, our data from the identified studies have prompted the question for further examination: is it the group experience, the reflection per se, or the subject focus of the reflection that mediates the “success” of an RP group? This question also needs further testing.

### 4.1. Limitations

The quality of the studies involving quantitative methodology varied widely; with only three of the nine rated as high-quality (score > 80% [16]). The variable quality of these largely data-linked cohort studies does raise the risk of bias and may limit the general applicability of the findings [50]. Notwithstanding this, some of the higher-quality studies emerged in relation to the effect of RP on empathy, potentially lending itself to a quantitative examination of this relationship with a meta-analysis, an opportunity missed by us but perhaps worthy of future studies.

Our strict inclusion and exclusion criteria might have limited our findings. For example, we highlighted the value of group settings for RP based on specific exclusion of solo RP practice, which might have its appeal for students constrained by the fear of exposure and lack of safe space. Moreover, in excluding RP studies involving communications skills (perceiving these as “technical skills”), we may have missed important studies pertaining to RP and empathy mediated by its effect on communication.

Finally, our review is susceptible to language and cultural bias, having excluded non-English papers.

### 4.2. Implications for Future Research

The notable absence of studies examining the relationship between RP and wellbeing, other than non-specific effects addressing loneliness and isolation, highlights an important area for future elucidation. Further, the lack of consistent or comprehensive assessment tools appears to be a consequence of the absence of conceptual or operational clarity regarding what really constitutes “success” or outcomes for these groups beyond empathy change. As far as we are aware, there are no studies examining outcomes related to wellbeing or operationalisation of professionalism.

## 5. Conclusions

Although tentative only, our findings illustrate the long-mooted value of group RP in medical student education. Perhaps the strongest evidence lies in its effect on promoting professionalism and empathy in medical students, being an important target for contemporary medical educators. Further, we have identified some clues as to when and how RP could be delivered, yet to be empirically tested. We join the call to bring to the fore the “hidden curriculum” in medicine and to continually refine and improve its delivery in medical education.

## Data Availability

The data collected for this review is available upon request from the corresponding author.

## Appendix A

**Table A1:** Included studies: characteristics, outcomes, and methodological quality.

Study Author/Year/Country	Research Aim/Question(s)	Participants Sample Size/Nature/ Settings	Description Study Design/Comparison Group(s), If Any	Outcomes	Methodological Quality Notes and Limitations	Level of Evidence OCEBM	Quantitative Rating	Qualitative Rating
Airagnes et al., 2014 [34] France	To examine changes in empathic abilities in an optional Balint groups	34 fourth-year medical students in an optional “doctor-patient relationship training” certificate	Quantitative methodology Measured interpersonal reactivity index (IRI) and emotional reactions (empathic-approach, rejecting-attitude, intellectual-interest and fear of emotion contagion) in response to two case-reports before the training sessions and 4 months later Compared with 129 participating in other certificates	An increase of IRI fantasy-scale (*p* = 0.02) and a decrease of IRI empathic-concern (*p* = 0.006) at follow-up, regardless of the group. Empathic-approach only increased in the “Balint group” and for the first case-report (*p* = 0.023), with a difference between the groups at follow-up (*p* = 0.003). Balint groups may enable students to better handle difficult clinical situations, e.g., those presented by patients with borderline personality traits.	Intervention group (“Balint group”) not described in details Lack of randomisation and scale justification which impact on the causality of conclusion Sample size not justified	2	77%	-
Bird et al., 2020 [22] United States	To cultivate resilience and promote wellness during students’ core clerkship rotations	144 clerkship students at two academic institutions (74 at University A; 70 at University B)	Both quantitative and qualitative data collected Learners completed pre- and post-curriculum surveys, including the Connor-Davidson Resilience Scale (CD-RISC; optional) Focus groups conducted with seven students at University A	Students valued connecting with peers and feeling less alone. The need to construct a setting conducive to comfortable reflection for all learners-not all students found these sessions necessary. Sessions may have improved resilience	Research question not clearly stated Subject characteristics not described Larger sample size but not justified CD-RISC fluctuation might subject to confounders which were not considered Reflexivity not considered	3	41%	C
Brand et al., 2016 [28] United Kingdom	To explore if photographs, narratives and small group collaborative dialogue fosters reflective learning, enhances reflective capacity and shift attitudes towards caring for older adults	128 (out of a cohort of 240) second year medical students; 95 students submitted an individual written reflection	Mixed method evaluation design, measuring attitudes using pre and post questionnaire responses and individual written reflections, exploring their perceptions toward older adults A 13-item validated Geriatric Attitude Scale was included.	Positive shifts in medical students’ perceptions towards older adults. The qualitative reflections were captured in four main themes: the opportunity provided to envision working with older adults; the tension created to challenge learners’ misinformed assumptions, and the work of dismantling those assumptions, leading to seeing older people as individuals	Small sample size No attempt to reach data saturation Thematic analysis described Reflexivity considered Conclusion supported by the results	3	86%	A
Chou et al., 2011 [23] United States	To describe a clerkship model called Veteran Affairs Longitudinal Rotations (VALOR), designed to establish a supportive learning environment for small peer groups	Seven groups of third year medical students (42 total) across three academic years, one hour per week during VALOR	Immediate post surveys and focus groups at the end of VALOR, and with follow-up surveys 5 to 27 months after completing VALOR	Students strongly valued support through clerkship challenges, meeting for facilitated reflection, appreciating patient experiences across the continuum of care, developing critical professional skills, and communication around patient care	Voluntary participation might skew the results No clear research question stated Some triangulation through both focus group and survey No reflexibility considered Confounder of concurrent clerkship activities not taken into account	-	-	C
Chu et al., 2018 [36] Taiwan	To determine psychosocial issues among patients and their family members through reflective dialogue groups	50 medical students were rotated to the department of Paediatrics for one month (7–9 on each rotation) Each student completed the reflective writing assignment and participated in one of the six group discussion sessions.	The recordings of the six reflective group sessions were transcribed for thematic analysis. A six-step theme generation process was conducted in the first reading stage of all transcripts by four researchers.	A total of 108 psychosocial issues were coded and categorized into six main themes: medical communication, the intricate medical ecological system, role and function of a family, development of medical professionalism, ethical dilemmas, and various patient perspectives from diverse cultural backgrounds. They illustrate that medical care should focus not only on illnesses but also patients’ psychosocial narratives	Clear aim of the study Sample size not justified Form of data recorded and transcribed with audit trail Using the content of the reflective groups for research analysis might introduce bias.	-	-	B
Duke et al., 2015 [24] United States	To analyse the effects of a professionalism course on empathy and self-reflection (two elements of professionalism) and their perceptions about the course	Third year medical students, meeting virtually throughout the year. 240 students who provided online feedback	Mixed methodology including the Groningen Reflection Ability Scale (GRAS) and the Jefferson Scale of Empathy (JSE) before and after the course and anonymous online feedback, which was analysed using thematic content analysis.	JSE demonstrated preservation of empathy rather than its decline. A statistically significant increase in GRAS scores (*p* < 0.001) This study supports previous findings showing that students benefit from peer groups and discussion in a safe environment, which may include the use of a virtual group video platform	Poor generalisability due to single institution involvement No clear research question stated Sample size not justified No reflexivity considered No subgroup analysis	3	82%	B
Gajree 2021 [29] United Kingdom	To assess whether a Balint group helped gain a better understanding of the role of emotions in the doctor–patient relationship	16 fourth or fifth year (of the 41 third, fourth and fifth year) medical students on clinical placement following their voluntary 5-week Balint groups	All completed an anonymous questionnaire following the final Balint group session about their experience. The questionnaire [4] entailed responding to a number of statements on a 5-point Likert scale and providing written feedback to open-ended questions about the group.	The groups helped students to think about the place of emotions in patient encounters, and the doctor-patient relationship. Most agreed that participating in a Balint group was an important part of their training as a doctor. Students overwhelmingly indicated that Balint groups provide an aspect of training that is not currently addressed elsewhere in the curriculum	Low number of participants reducing its generalisability The potential for selection bias due to its voluntary nature No reflexivity considered Clear statement of aim	-	-	B
Gold et al., 2019 [25] United States	To create a safe space to regularly discuss shared experiences in medical school while providing a near peer opportunity for psychiatry residents to acquire group facilitation experience	30 students participated in groups led by psychiatry residents 18 completed post-surveys in first- and second-year medical students attending voluntary, biweekly support groups	Surveys at baseline and 6 months included qualitative assessments of groups and validated surveys to assess empathy, wellness, and loneliness. Separate surveys assessed attrition. Statistical analyses (descriptive statistics) and thematic analysis	Groups may benefit in improving impostor syndrome and connection with others (decreased loneliness), allowing exposure to and tolerance of diverse perspectives, increasing insight into the importance of self-care and emotional self-awareness, allowing practice for collaborative skills, and increasing thoughtful approaches to patient care	Unclear objective Study design inappropriate–hypothesis driven Unclear sampling strategy Reflexivity identified as limitation Study participants’ characteristics not described Lack of control group Voluntary participation might skew results	5	27%	C
Imperato et al., 2021 [26] United States	To analyse what structured Reflection Rounds had on self-reported empathy and emotional intelligence scores	285 voluntary third-year medical students during their core clinical clerkships Small-group meetings, where students reflect upon their thoughts, feelings, and emotions about clinical experiences and receive feedback from peers and a trained facilitator	Quantitative measures of pre- and post-intervention utilizing the self-reported Jefferson scale of empathy (JSE) student version and Wong law emotional intelligence scale (WLEIS)	Empathy scores increased from 80.4 to 82.6 (*p* = 0.02) post-intervention. No significant difference in EI scores was demonstrated post-intervention, 5.4 to 5.5 (*p* = 0.55)	Lack of a control group No justification of sample size Lack of compliance with consistent utilization of unique identification coding precluded individual analysis of matched data	3	73%	-
Lemogne et al., 2020 [35] France	To assess the effects of Balint groups and narrative medicine training on clinical empathy	362 out of the 392 fourth year medical students completed The intervention groups received either seven sessions of 1.5-h Balint groups or a 2-h lecture and five sessions of 1.5-h narrative medicine training	117 fourth-year medical students in the control group, 125 in the Balint group and 120 in the narrative medicine group The main quantitative outcome was the change in JSPE-MS© score from baseline to one week after the last session.	Adjusting for participants’ characteristics at baseline, Balint groups remained associated with better outcomes compared to the control group (beta = 2.673, *p* = 0.030) Balint groups may promote clinical empathy to some extent among medical students, at least in the short run	Clear aim and objective Appropriate study design Confounding considered with control and comparison groups Underpower between the two intervention groups to draw conclusions as regards the lack of difference self-reported measures may be more influenced by social desirability biases than objective measures	2	93%	-
Lutz et al., 2017 [38] Germany	To explore both the attitudes of those students towards the program and factors that might hinder or enhance how students engage in reflective discourse	Of the 168 contacted preclinical students who attended a group mentoring program, 14 consented to participating in the focus group interviews. Eight mentors and one co-mentor agreed to participate in individual interviews.	A qualitative design was applied using semi-structured focus group interviews with preclinical students and semi-structured individual interviews with mentors and co-mentors	Some students valued the new program and named positive outcomes regarding several features of professional development and enriching experiences. Others expressed aversive attitudes: unclear goals and benefits, interpersonal problems within the groups hindering development and intrapersonal issues such as insecurity and traditional views of medical education	Clear research question Appropriate and clear sampling strategy Reflexivity considered Triangulation from different sources attempted One setting attitude analysis limited the generalizability The students who agreed to take part in the interviews may have been particularly motivated and reflective	-	-	A
McManus et al., 2020 [39] Ireland	To establish and evaluate the impact of a 6-week Balint group on empathy and resilience during psychiatry rotation	28 out of the 50 eligible fourth-year medical students	A prospective study used the Jefferson Scale of Empathy–Student Version and the Brief Resilience Scale before and after 6-week Balint groups One week after the final Balint group in each course, the Balint lead and co-lead met with the participants to conduct a focus group	Enthusiasm regarding the value of Balint groups in promoting self-reflection and gaining insight into self- and patient-care dynamics. There was a significant difference in empathy scores pre- and post-Balint intervention. There was no significant difference in resilience scores	Clear study aims and design No control group limiting generalisability Sample size is relatively small Confounding variables such as whether lectures and practical teaching in psychiatry could have improved empathy or whether impending exams could have affected post-group scores in resilience or empathy	4	77%	B
O’Neill et al., 2016 [31] Australia	To pilot and employ the traditional method pioneered by Michael and Enid Balint for general practitioners working in London after the Second World War	One group of six third-year graduate students, meeting weekly over six weeks.	Evaluation includes pre- and post-questionnaires, a 1000-word essay and leaders’ observations.	Traditional Balint method needs to be modified for students at a point in their training where they have not yet been exposed to patients for long enough to develop meaningful patient relationships. Some evidence of a heightened awareness of the dynamics of doctor–patient relationships and the importance of psychological/emotional factors (including their own prejudices) Balint-style groups could be an effective way of encouraging reflection on the importance of emotions in the doctor–patient relationship.	Small sample size Recall bias: notes are memory-dependent and may be influenced by the leaders’ own (unconscious) prejudices and interpretations procedural change introduced after the third session No baseline measurement	-	-	C
Parker et al., 2012 [32] Australia	To examine whether groups could be effectively implemented within the curriculum and whether the student participants would value such an opportunity	Ten third-year medical students during six of the eight weeks of their clinical rotation in psychiatry Two rotations have been completed	The educational value of each meeting and the group overall is assessed using questionnaires.	Students were less certain of the relevance to their clinical practice, which requires adaptation of method and process to context Short-term clinical reflection groups can be effectively implemented for medical students in a hospital environment, supporting students in the process of learning to work in doctor-patient relationships	No comparison group Small sample size limiting its generalisability. No reflexivity considered No clear analysis methodology	-	-	C
Parker et al., 2014 [33] Australia	To consider whether clinical reflection groups following the Balint process would be perceived as useful and relevant to the learning needs of the student participants.	All group members (*n* = 42) medical students in psychiatry at a large Australian tertiary referral hospital This paper presents the outcomes of the named project at the conclusion of its one-year trial [32].	A grounded theory approach, including survey data, thematic analysis of written feedback, and facilitator reflection on the process.	The evaluation suggested that fidelity of the Balint group experience was achieved and that student attitudes were neutral to mildly positive regarding the educational experience. Balint groups are potentially useful to medical students in the Australian context. More useful when applied in less alienating learning environments, such as during general practice rotations.	Larger sample size compared to previously presented data [32] No reflexivity considered Tape recording and triangulation of multiple sources of data attempted	-	-	B
Plack et al., 2010 [27] United States	To determine whether peer-facilitated virtual action learning (VAL) demonstrated reflection and critical thinking around complex issues during their paediatric clerkship	70 clerkship students were introduced to reflection and participated in VAL by using an electronic discussion board 70 critical incidents, 210 discussion board entries, 70 revised critical incidents, and 70 reflective essays were analysed	Qualitative methods were used to analyse initial critical incidents, discussion threads, and summative reflective essays. Two non-physician researchers involved in data analysis helped design the study but were not involved in its implementation.	Broadened perspectives (44/70), questioned assumptions (12/70), and reconfirmed thinking (14/70). Content themes included communication, role identification, medical treatment concerns, and limited voice and power. Most students demonstrated reflection on complex clinical issues. Themes portrayed struggles encountered and exposed issues in the hidden curriculum, suggesting a lack of voice and power that may lead to missed learning opportunities	Small sample of convenience No baseline measures or use of a control group Triangulation of several written data sources Analysis done by separate investigators Reflexivity considered	-	-	A
Wen et al., 2015 [37] Taiwan	To investigate the characteristics of feedback in a reflective dialogue group, involving a structured narrative reflective writing combined with pluralistic group discussion with a tutor and peers	40 fifth-year medical students of five monthly interval rotations at the paediatric department of a medical centre in eastern Taiwan	The evaluative questionnaire regarding the benefits of reflection with others was administrated following the group session	Each student attained 1.25 times the depth or breadth of reflection after receiving feedback and experienced the benefits of reflection with others. the medical students had time to think deeply and broadly about psychosocial issues among patients and their family members. Facilitative feedback providing new knowledge, deeper discussion, and exploring new ways of action planning for psychosocial issues was recommended to promote students’ reflective capacity.	Clear research question and aim Context and setting adequately described Sample size not justified Triangulation of different data sources with systematic transcription and subsequent analysis Reflexivity not considered	-	-	B
Yakeley et al., 2011 [30] United Kingdom	To evaluate the effectiveness of two psychodynamic psychotherapy teaching methods, student psychotherapy scheme (SPS) and Balint group, on doctor-patient communication and the doctor-patient relationship	28 (out of the 49 volunteered, who were subsequently interviewed for suitability) first-year clinical medical students	Randomised controlled trial of three groups, 10 students each (SPS group, Balint group starting at baseline and Balint group starting at 3 months-acting as partial controls) They were rated on a questionnaire testing their knowledge of emotional and psychodynamic aspects of the doctor-patient relationship administered at baseline, at 3 months and at 1 year	At 3 months, students in the SPS and Balint groups scored higher than the partial control group, the difference approaching significance at the 5% level. At 1 year, participation in either teaching method led to significantly higher scores compared with baseline. Psychodynamic psychotherapy teaching methods are effective in increasing students’ knowledge of the doctor-patient relationship and potentially also improving their communication skills	No control group at 1 year The small number of students involved and the low number of questionnaires returns at 3 months underpower the analysis Unvalidated measure of communication skill No attempt on investigator blinding	3	77%	-
